# RBC transfusion and necrotizing enterocolitis in very preterm infants: a multicenter observational study

**DOI:** 10.1038/s41598-024-64923-7

**Published:** 2024-06-21

**Authors:** Dan Dang, Xinyue Gu, Siyuan Jiang, Wenli Li, Wenhao Zhou, Yun Cao, Shoo Kim Lee, Hui Wu, Jianguo Zhou, Chinese Neonatal Network (CHNN), Xiuyong Chen, Huyan Zhang, Xiuying Tian, Jingyun Shi, Zhankui Li, Chuanzhong Yang, Ling Liu, Zuming Yang, Jianhua Fu, Yong Ji, Dongmei Chen, Changyi Yang, Rui Chen, Xiaoming Peng, Ruobing Shan, Shuping Han, Hui Wu, Lili Wang, Qiufen Wei, Mingxia Li, Yiheng Dai, Hong Jiang, Wenqing Kang, Xiaohui Gong, Xiaoyun Zhong, Yuan Shi, Shanyu Jiang, Bing Sun, Long Li, Zhenlang Lin, Jiangqin Liu, Jiahua Pan, Hongping Xia, Xiaoying Li, Falin Xu, Yinping Qiu, Li Ma, Ling Yang, Xiaori He, Yanhong Li, Deyi Zhuang, Qin Zhang, Wenbin Dong, Jianhua Sun, Kun Liang, Huaiyan Wang, Jinxing Feng, Liping Chen, Xinzhu Lin, Chunming Jiang, Chuan Nie, Linkong Zeng, Mingyan Hei, Hongdan Zhu, Hongying Mi, Zhaoqing Yin, Hongxia Song, Hongyun Wang, Dong Li, Yan Gao, Yajuan Wang, Liying Dai, Liyan Zhang, Yangfang Li, Qianshen Zhang, Guofang Ding, Jimei Wang, Xiaoxia Chen, Zhen Wang, Zheng Tang, Xiaolu Ma, Xiaomei Zhang, Xiaolan Zhang, Fang Wu, Yanxiang Chen, Ying Wu, Joseph Ting

**Affiliations:** 1 Department of Neonatology, Children’s Medical Center, First Hospital of Jilin University, No. 1 Xinmin Street, Changchun, 130021 Jilin China; 2 Department of Neonatology, Children’s Hospital of Fudan University, 399 Wanyuan Road, Minhang District, Shanghai, 201102 China; 3 Department of Neonatology, The Third Affiliated Hospital of Zhengzhou University, Zhengzhou, Henan China; 4 Department of Pediatrics, Maternal-Infant Care Research Centre, Mount Sinai Hospital, University of Toronto, Toronto, ON Canada; 5 Children’s Hospital of Fudan University, Shanghai, China; 6 Children’s Hospital of Zhejiang University School of Medicine, Zhejiang, China; 7 The Third Affiliated Hospital of Zhengzhou University, Zhengzhou, China; 8 Guangzhou Women and Children’s Medical Center, Guangzhou, China; 9 Tianjin Obstetrics & Gynecology Hospital, Tianjin, China; 10 Gansu Provincial Maternity and Child Care Hospital, Lanzhou, China; 11 Northwest Women’s and Children’s Hospital, Xi’an, China; 12 Shenzhen Maternity and Child Health Care Hospital, Shenzhen, China; 13 Guizhou Women and Children’s Hospital, Guiyang, China; 14 Suzhou Municipal Hospital Affiliated to Nanjing Medical University, Nanjing, China; 15 Shengjing Hospital of China Medical University, Shenyang, China; 16 Children’s Hospital of Shanxi, Taiyuan, China; 17 Quanzhou Women and Children’s Hospital, Quanzhou, China; 18 Fujian Women and Children’s Medical Center, Guangzhou, China; 19 Children’s Hospital of Nanjing Medical University, Nanjing, China; 20 Hunan Children’s Hospital, Changsha, China; 21 Qingdao Women and Children’s Hospital, Qingdao, China; 22 Nanjing Maternity and Child Health Care Hospital, Nanjing, China; 23 The First Bethune Hospital of Jilin University, Changchun, China; 24 The First Affiliated Hospital of Anhui Medical University, Hefei, China; 25 Women and Children’s Hospital of Guangxi Zhuang Autonomous Region, Nanning, China; 26 The First Affiliated Hospital of Xinjiang Medical University, Ürümqi, China; 27 Foshan Women and Children’s Hospital, Foshan, China; 28 The Affiliated Hospital of Qingdao University, Qingdao, China; 29 Henan Children’s Hospital, Zhengzhou, China; 30 Children’s Hospital of Shanghai, Shanghai, China; 31 Chongqing Health Care Center for Women and Children, Chongqing, China; 32 Children’s Hospital of Chongqing Medical University, Chongqing, China; 33 Wuxi Maternity and Child Healthcare Hospital, Wuxi, China; 34 Children’s Hospital of Soochow University, Suzhou, China; 35 People’s Hospital of Xinjiang Uygur Autonomous Region, Ürümqi, China; 36 Yuying Children’s Hospital Affiliated to Wenzhou Medical University, Wenzhou, China; 37 Shanghai First Maternity and Infant Hospital, Shanghai, China; 38 Anhui Provincial Hospital, Hefei, China; 39 Xinhua Hospital Affiliated to Shanghai Jiaotong University School of Medicine, Shanghai, China; 40 Qilu Children’s Hospital of Shandong University, Dezhou, China; 41 The First Affiliated Hospital of Zhengzhou University, Zhengzhou, China; 42 General Hospital of Ningxia Medical University, Yinchuan, China; 43 Hebei Children’s Hospital, Shijiazhuang, China; 44 Hainan Women and Children’s Hospital, Haikou, China; 45 The Second Xiangya Hospital of Central South University, Changsha, China; 46 Ningbo Women & Children Hospital, Ningbo, China; 47 Xiamen Children’s Hospital, Xiamen, China; 48 Shaanxi Provincial People’s Hospital, Xi’an, China; 49 The Affiliated Hospital of Southwest Medical University, Luzhou, China; 50 Shanghai Children’s Medical Center Affiliated to Shanghai Jiaotong University School of Medicine, Shanghai, China; 51 First Affiliated Hospital of Kunming Medical University, Kunming, China; 52 Changzhou Maternal and Children Health Care Hospital, Changzhou, China; 53 Shenzhen Children’s Hospital, Shenzhen, China; 54 Jiangxi Provincial Children’s Hospital, Nanchang, China; 55 Xiamen Maternity and Child Health Care Hospital, Xiamen, China; 56 Zhuhai Center for Maternal and Child Health Care, Zhuhai, China; 57 Guangdong Women and Children’s Hospital, Guangzhou, China; 58 Wuhan Children’s Hospital, Wuhan, China; 59 Beijing Children’s Hospital of Capital Medical University, Beijing, China; 60 Maternal and Children Hospital of Shaoxing, Shaoxing, China; 61 The First People’s Hospital of Yunnan Province, Kunming, China; 62 Dehong People’s Hospital of Yunnan Province, Dehong, China; 63 First Affiliated Hospital of Xian Jiaotong University, Xi’an, China; 64 Inner Mongolia Maternal and Child Health Care Hospital, Xilinhot, China; 65 Dalian Municipal Women and Children’s Medical Center, Dalian, China; 66 Lianyungang Maternal and Children Health Hospital, Lianyungang, China; 67 Children’s Hospital Affiliated to Capital Institute of Pediatrics, Beijing, China; 68 Anhui Children’s Hospital, Hefei, China; 69 Fuzhou Children’s Hospital of Fujian Province, Fuzhou, China; 70 Kunming Children’s Hospital, Kunming, China; 71 Shenzhen Hospital of Hongkong University, Shenzhen, China; 72 Peking Union Medical College Hospital, Beijing, China; 73 Obstetrics & Gynecology Hospital of Fudan University, Shanghai, China; 74 The Affiliated Hospital of Guizhou Medical University, Guizhou, China; 75 Qinghai Women and Children Hospital, Qinghai, China; 76 The International Peace Maternity & Child Health Hospital of China Welfare Institute, Shanghai, China; 77 Children’s Hospital of Zhejiang University, Zhejiang, China; 78 Inner Mongolia People’s Hospital, Hohhot, China; 79 Xiamen Humanity Hospital, Xiamen, China; 80 Shanghai General Hospital, Shanghai, China; 81 The First People’s Hospital of Yinchuan, Yinchuan, China; 82 The Third Hospital of Nanchang, Nanchang, China; 83 University of Alberta, Alberta, Canada

**Keywords:** Preterm infants, Transfusion-associated necrotizing enterocolitis, Red blood cell transfusion, Prognosis, Risk factors, Infant necrotizing enterocolitis

## Abstract

The causal relationship between Packed red blood cell (RBC) transfusion and necrotizing enterocolitis (NEC) remains uncertain. This study aims to provide an exploration of transfusion and NEC in very preterm infants. Using data from the Chinese Neonatal Network cohort study between 2019 and 2021, the analysis focused on very preterm infants (with a birth weight of < 1500 g or a gestational age of < 32 weeks) who developed NEC after receiving transfusions. The time interval between the prior transfusion and NEC was analyzed. An uneven distribution of the time interval implies an association of transfusion and NEC. Additionally, multivariable logistic analysis was conducted to detect the prognosis of defined transfusion-associated NEC(TANEC). Of the 16,494 infants received RBC transfusions, NEC was noted in 1281 (7.7%) cases, including 409 occurred after transfusion. Notably, 36.4% (149/409) of post-transfusion NEC occurred within 2 days after transfusion. The time interval distribution showed a non-normal pattern (Shapiro–Wilk test,* W* = 0.513, *P* < 0.001), indicating a possible link between transfusion and NEC. TANEC was defined as NEC occurred within 2 days after transfusion. Infants with TANEC had a higher incidence of death (adjusted OR 1.69; 95% CI 1.08 to 2.64), severe bronchopulmonary dysplasia (adjusted OR 2.03; 95% CI 1.41 to 2.91) and late-onset sepsis (adjusted OR 2.06; 95% CI 1.37 to 3.09) compared with infants without NEC after transfusion. Unevenly high number of NEC cases after RBC transfusions implies transfusion is associated with NEC. TANEC is associated with a poor prognosis. Further research is warranted to enhance our understanding of TANEC.

## Introduction

Necrotizing enterocolitis (NEC) is a frequently encountered gastrointestinal disorder that significantly contributes to adverse outcomes in premature infants. The etiologies of NEC encompass formula feeding, hypoxia, infection^1^. However, the debate over whether red blood cell (RBC) transfusion is associated with NEC in preterm infants continues to be widely discussed^2^.

Despite decades of extensive research, the relationship between blood transfusion and NEC remains elusive. Studies have conflicting findings^3–11^, with some suggesting RBC transfusion as a cause of intestinal injury, others proposing it as protective against NEC, and some finding no link. These evidences supporting this association is of lower quality, primarily consisting of case–control studies, with only a limited number of cohort studies conducted thus far. Additionally, the restricted sample sizes across all these studies pose a challenge to definitively clarify the connection between transfusion and NEC. Does transfusion-associated necrotizing enterocolitis (TANEC) exist, and if so, at what critical time point does it occur? Furthermore, what is the prognosis of infants affected by TANEC? A more comprehensive understanding of association between transfusion and NEC is urgently needed and will greatly benefit disease prevention and prognosis.

This study, based on data from the Chinese Neonatal Network (CHNN) cohort study involving very premature infants, seeks to establish a causal link between transfusions and NEC. Additionally, it aims to investigate the prognosis of TANEC infants. The study will provide a more detailed description of TANEC and serve as a basis for disease prevention and treatment.

## Methods

### Study design

This retrospective study conducts a secondary analysis of the CHNN database. Established in 2018, this collaborative network encompasses 79 (by 2021) neonatal intensive care units (NICUs) across China. The network collects clinical data on infants with a birth weight of < 1500 g or a gestational age of < 32 weeks from member hospitals^12^. The study was undertaken with the approval from the ethics review board of the Children’s Hospital of Fudan University (2018–296), the central hub of CHNN. Written informed consent was not required for retrospective observational study as per the [Ethics Review Board of the Children’s Hospital of Fudan University]. All methods in this study were performed in accordance with the relevant guidelines and regulations.

### Study population

This study utilized the CHNN database to identify all infants who received RBC transfusions between January 2019 and December 2021. Transfusion strategy was implemented in accordance with the "Guideline for Pediatric Transfusion" issued by the National Health Commission of the People's Republic of China^13^. Infants with major congenital anomalies or missing data regarding NEC or receipt of RBC transfusions were excluded from the analysis.

### Definitions

NEC was defined as Bell’s Stage II or greater according to an established criteria^14^. Spontaneous Intestinal Perforation (SIP) refers to individuals diagnosed intraoperatively or imaging suggestive of intestinal perforation lacking clinical features of necrotizing enterocolitis (NEC) is not included in the NEC group. Infants who received transfusions but did not develop NEC during hospitalization were categorized “No-NEC”. Infants with NEC were segregated into post-transfusion NEC, pre-transfusion NEC and Undefined NEC (Ud-NEC) based on the sequence of NEC and transfusion. Ud-NEC referred to cases where transfusion and NEC occurred on the same day and the onset of NEC (before or after transfusion) was unclear. Among the post-transfusion NEC, NEC that occurred within two calendar days after transfusion exposure was defined as TANEC, NEC cases that occurred beyond 2 days post transfusion, along with pre-transfusion NEC cases, were considered unrelated to transfusion and defined as UNTA-NEC. The time interval between transfusion and NEC refers to the period between the most recent transfusion and the onset of NEC. The accumulated number of transfusions refers to the total count of transfusions administered before TANEC.

### Variables

Trained data abstractors obtained variables from the neonate's medical records^15^. The data were then transmitted electronically to central hub of CHNN, maintaining patient anonymity. At each site, site investigators were accountable for data quality control. Routine data auditing, and periodic feedback were provided to each site to ensure data quality ^16^.

Clinical characteristics of TANEC infants were detailed including maternal age, cesarean delivery, birth weight, gestational age by best obstetric estimate, sex, small for gestational age(SGA, birth weight < 10th percentile for the gestational age according to the Chinese neonatal birth weight values^17^), placenta transfusion including delayed cord clamping and umbilical cord milking, low 5-min Apgar score (Apgar score ≤ 7), endotracheal intubation during resuscitation, inborn, placental transfusion, age at the 1st feed and duration of fasting and antibiotic therapy in first seven days of life. Age at transfusion was defined as the postnatal days of life (DOL) and postmenstrual age (PMA).

The outcomes of TANEC infants included cystic periventricular leukomalacia (PVL), severe retinopathy of prematurity (ROP), late-onset sepsis (LOS), severe bronchopulmonary dysplasia (BPD), and mortality. Cystic PVL was defined as the presence of periventricular cysts on cranial ultrasound or magnetic resonance imaging. Severe ROP was defined as ROP stage ≥ 3 according to the International Classification of ROP^18^. LOS was defined as culture-proven sepsis between 3 and 28 calendar days of life. Severe BPD was defined as nasal continuous positive airway pressure or intermittent positive pressure ventilation or invasive ventilation requirement at 36 weeks of PMA or at discharge, transfer or death if before 36 weeks corrected gestational age^19^.

### Statistical analysis

Data analyses were conducted using SAS version 9.4 (SAS Institute; Cary, NC, USA). Categorical variables were presented as frequencies and percentages, and group comparisons were performed using the chi-square test. Non-normally distributed data were presented as medians and quartiles, and group comparisons were performed using the Wilcoxon test. The normality of the time interval was assessed using the Shapiro–Wilk test. If *W* = 1 and *P* > 0.05, it suggests a normal distribution. Otherwise, a non-normal distribution is indicated. Multiple logistic regression analysis was performed to determine the odds ratio (OR) for TANEC for each clinical factor, with adjustment for confounding variables that showed baseline imbalances among the groups. Statistical significance was defined as a two-tailed *P* < 0.05.

### Ethical approval

Ethics review board of the Children’s Hospital of Fudan University (2018-296) and all participating hospitals.

## Results

### Characteristics of included cases

**Figure 1:**
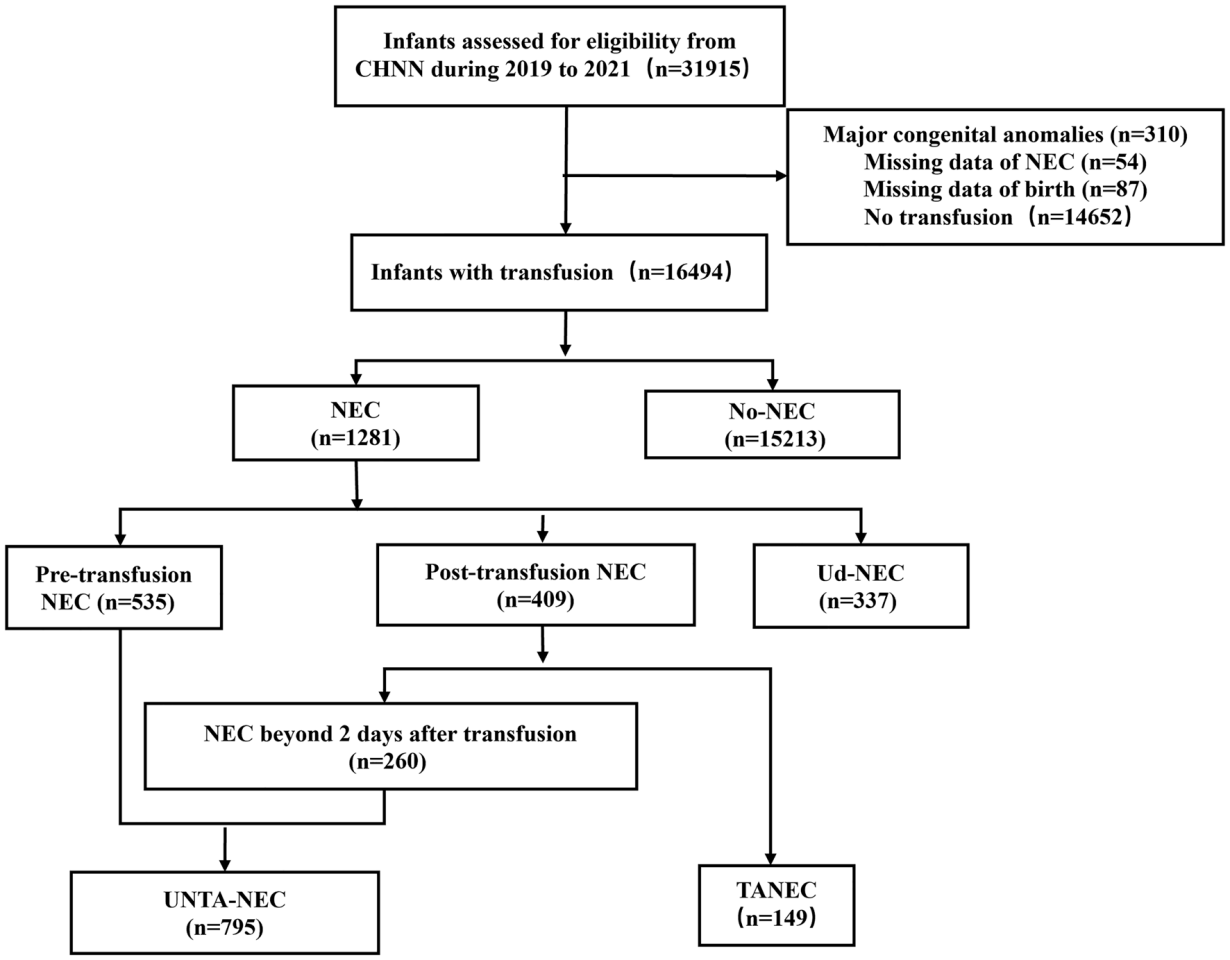
Flow chart of preterm infants with transfusion. *CHNN* Chinese Neonatal Network, *NEC* necrotizing enterocolitis, *No-NEC* infants without NEC, *UNTA-NEC* preterm infants with NEC prior to all transfusion or NEC occurred beyond 2 days after transfusion, *TANEC* NEC occurred within 2 days after transfusion, *Ud-NEC* undefined NEC was defined as NEC that transfusion and NEC occurred on the same day and the onset of NEC (before or after transfusion) was ambiguous.

Between Jan 2019 and Dec 2021, a total of 31915 very preterm infants with a gestational age < 32 weeks or a birth weight < 1500 g were identified. Among 16,494 patients who received RBC transfusions during their hospitalization, we recorded a total of 41,973 transfusion episodes and 1281 cases of NEC (7.7% as opposed to 4.9% across all infants) were recorded. Of all NEC cases, there were 535 pre-transfusion NEC, 337 Ud-NEC and 409 post-transfusion NEC (Fig. 1).

### Distribution of time intervals between transfusion and NEC

**Figure 2:**
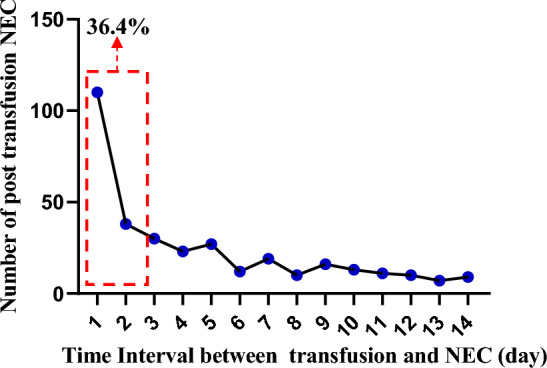
Distribution of time interval between transfusion and NEC. *NEC* necrotizing enterocolitis.

The distribution curve of time intervals between transfusion and NEC was plotted. Of the 409 post-transfusion NEC cases identified, 149 (36.4%) occurred within 2 days after transfusion, 201 (49.1%) within 3 days, 224 (54.8%) within 4 days, and 282 (68.9%) within one week. With an increase in the time elapsed after transfusion, there was a reduction in the occurrence of NEC (Fig. 2 and Table S1). The time interval between transfusion and the presentation of NEC exhibits a non-normal distribution (Shapiro–Wilk test; *W* = 0.513, *P* < 0.001), suggesting an association between transfusion and NEC.

### Prognosis of TANEC infants compared with No-NEC or UNTA-NEC infants

**Figure 3:**
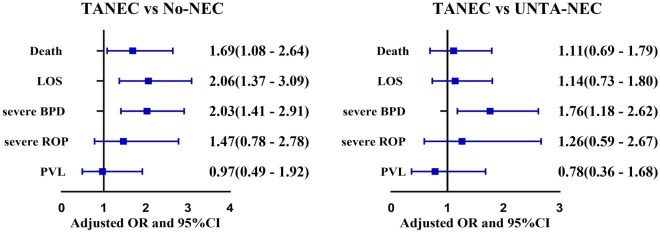
Outcomes of TANEC infants. *BPD* bronchopulmonary dysplasia, *ROP* retinopathy of prematurity,* PVL* periventricular leukomalacia. TANEC vs No-NEC: adjusted for gestational age, placental transfusion, age at first feed in days ≤ 3, PDA; TANEC vs UNTA-NEC: adjusted for gestational age, endotracheal incubation during resuscitation, age at first feed in days ≤ 3, inborn, low 5-min Apgar score ≤ 7, duration of antibiotic therapy in 1st 7 days of life in days > 4.

Characteristics of infants of TANEC, UNTA-NEC, and No-NEC are shown in Table 1. TANEC primarily occurred between DOL15 to 42 (60.4% of all TANEC, Table S2) or at 31 to 34 weeks of PMA (59.7% of all TANEC, Table S3). By comparing with No-NEC, infants of TANEC exhibited a higher mortality rate (adjusted OR 1.69; 95% CI 1.08 to 2.64; *P* = 0.022), a higher occurrence of severe BPD (adjusted OR 2.03; 95% CI 1.41 to 2.91; *P* < 0.001) and more frequent LOS (adjusted OR 2.06; 95% CI 1.37 to 3.09; *P* < 0.001). When compared with UNTA-NEC, TANEC infants still showed a higher rate of severe BPD (OR 1.76; 95% CI 1.18 to 2.62; *P* = 0.006) (Fig. 3 and Table S4).

**Table 1:** Characteristics of TANEC infants.

	TANEC (N = 149)	No-NEC (N = 15,213)	UNTA-NEC (N = 795)	*P* values	
				TANEC vs No-NEC	TANEC vs UNTA-NEC
Maternal age (median, IQR)	30 (28–34)	31 (28–34)	31 (28–34)	0.587	0.905
Cesarean delivery (n, %)	78/149 (52.4)	8900/15,167 (59.7)	482/794 (60.7)	0.119	0.057
Gestational age, weeks (median, IQR)	28.9(27.1–30.4)	29.4(28–30.9)	29.9 (28.3–31)	0.003	< 0.001
Gestational age weeks (n, %)					
< 26	14/149 (9.4)	945/15,213 (6.2)	47/795(5.9)	0.043	0.052
26–28	43/149 (28.9)	3137/15,213 (20.)	130/795(16.4)	0.017	< 0.001
29–30	45/149 (30.2)	5545/15,213 (36.5)	286/795 (36.0)	0.518	0.543
30–32	43/149 (28.9)	4570/15,213 (30.0)	274/795 (34.5)	0.828	0.538
> 32	4/149(2.7)	1016/15,213 (6.7)	58/795 (7.3)		
Birth weight, grams (median, IQR)	1140 (950–1310)	1200 (1004–1400)	1240 (1030–1450)	0.001	< 0.001
Birth weight, grams (n, %)					
< 1000	47/149 (31.5)	3343/15,213(22.0)	160/795 (20.1)	0.002	< 0.001
1000–1500	87/149 (58.4)	9453/15,213 (62.1)	482/795 (60.6)	0.936	0.754
≥ 1500	15/149 (10.1)	2417/15,213 (15.9)	153/795 (19.3)		
Male gender (n, %)	84/149 (56.4)	8597/15,207 (56.5)	464/794 (58.4)	0.984	0.697
SGA (n, %)	23/149 (15.4)	2270/15,133 (15.0)	127/794 (16.0)	0.881	0.866
Inborn (n, %)	103/149 (69.1)	9392/15,213 (61.7)	474/795 (59.6)	0.066	0.030
Placental transfusion (n, %)	59/149 (39.6)	4547/15,213 (29.9)	276/795 (34.7)	0.011	0.254
Endotracheal incubation during resuscitation (n, %)	54/149 (36.2)	4876/15,213 (32.1)	176/795 (22.1)	0.276	< 0.001
Low 5-min Apgar score (n %)	35/149 (23.5)	3367/15,213 (22.1)	122/795 (15.4)	0.691	0.014
Age at first feed in days ≤ 3 (n, %)	110/149 (73.8)	9862/15,213 (64.8)	494/795 (62.1)	0.023	0.006
Duration of antibiotic therapy in 1st 7 days of life in days > 4 (n, %)	101/149 (67.8)	9888/15,213 (65.0)	443/795 (55.7)	0.478	0.007
PDA (n %)	86/149 (58.5)	7352/15,213 (49.3)	396/795 (49.8)	0.026	0.083
PDA (required treatment) (n %)	39/149 (26.2)	3013/15,213 (19.8)	160/795 (20.1)	0.057	0.101

*NEC* necrotizing enterocolitis, *No-NEC* infants without NEC, *UNTA-NEC* preterm infants with NEC prior to all transfusion or NEC occurred beyond 2 days after transfusion, *TANEC* occurred within 2 days after transfusion, *SGA* small for gestational age.

## Discussion

This hospital-based, large-scale, multicenter observational study, featuring the largest sample size to date, unveils that more than one-third of post-transfusion NEC manifest within 2 days following transfusion. Furthermore, TANEC infants are often associated with increased mortality, higher rates of severe BPD and LOS.

The association between RBC transfusion and NEC has consistently been a controversial topic. Some studies assert RBC transfusion as a cause of intestinal injury^3–6^, while others have proposed that transfusion may serve as a protective factor against NEC^7,8^ or has no link with NEC^9,10^, and that anemia may pose as an independent risk factor for TANEC^11^. Recent bench studies have uncovered some evidence supporting TANEC. For example, researches have demonstrated that changes in mesenteric blood flow velocity, reoxygenation, and reperfusion after transfusion could incite intestinal oxidative stress injury^20–22^. Furthermore, multiple immune factors such as IL-1β, IL-6, IFN-γ, and ICAM-1 have been shown to increase in circulation following RBC transfusion^23,24^. Studies have described a murine model of NEC instigated by transfusions after anemia, illustrating typical NEC-like gut injuries in the anemia-transfusion group within 48 h post transfusion due to Toll-like receptor-4-mediated injury and intestinal epithelial barrier dysfunction^25,26^. Considering our data in tandem with prior animal studies, it is plausible that RBC transfusion is associated with NEC.

Definitive definition of TANEC varied in previous studies, with presenting variable periods ranging from 48 h to 7 days^11,27,28^. In this study, we observed that 36.9% of post-transfusion NEC cases occurred within the first 2 days after transfusion, with a marked decrease in incidence thereafter. This finding provides more evidence to support the definition of TANEC as NEC occurring within 48 h following RBC transfusion.

To mitigate the occurrence of TANEC, it is crucial to identify the high-risk population and delineate specific transfusion characteristics associated with TANEC. Our study suggests that very preterm infants at 15–42 DOL or at 31–34 weeks may be more susceptible to developing TANEC, and thus could potentially benefit from preventive measures such as considering withholding feeding during transfusions^29,30^. It is also possible that the underlying cause is the high incidence of anemia during this period, which requires more blood transfusions.

This large retrospective study provides detailed characteristics of transfusions associated with a higher risk of NEC, however, it is important to acknowledge several limitations. First, the retrospective design of the study precluded the analysis of potential factors such as hemoglobin levels, the volume of transfusion, and feeding volume during transfusion. Fortunately, all member units of CHNN adhere to the "Guideline for Pediatric Transfusion" issued by the National Health Commission of the People's Republic of China, thereby effectively mitigating the impact of these factors to the maximum extent possible. Second, the exclusion of transfusion episodes without a definite time interval with Ud-NEC may have resulted in selection bias, although this was necessary for accurate patient grouping. Nevertheless, the study's findings remain robust.

## Conclusions

In summary, we have observed an association between RBC transfusion and NEC in very premature infants, as evidenced by a significant increase in NEC occurrence within 2 days post transfusion. TANEC is also associated with higher risks of mortality, BPD and LOS. An in-depth comprehension of the characteristics concerning transfusion episodes linked to NEC may offer a robust theoretical underpinning for the standardization of clinical transfusion practices and the subsequent mitigation of NEC (Supplementary Information).

### Supplementary Information

Supplementary Tables.

## Data Availability

The authors declare that all data supporting the findings of this study are available within the article and its supplementary information files.
