# Supplementary material for: RBC transfusion and necrotizing enterocolitis in very preterm infants: a multicenter observational study
Source: Sci Rep. 2024 Jun 21;14:14345. doi: 10.1038/s41598-024-64923-7 (PMC11192881; doi:10.1038/s41598-024-64923-7)
Supplement: Supplementary file 1 — Supplementary Tables. [file 41598_2024_64923_MOESM1_ESM.docx]

**Table S1 Distribution of time intervals between transfusion and NEC**

| Time interval between the latest transfusion and NEC | NEC cases |
| --- | --- |
| 1 | 110 |
| 2 | 39 |
| 3 | 30 |
| 4 | 23 |
| 5 | 27 |
| 6 | 12 |
| 7 | 19 |
| 8 | 11 |
| 9 | 16 |
| 10 | 13 |
| 11 | 11 |
| 12 | 10 |
| 13 | 7 |
| 14 | 9 |
| 15 | 12 |
| 16 | 8 |
| 17 | 9 |
| 18 | 10 |
| 19 | 6 |
| 20 | 3 |
| 21 | 2 |
| 22 | 1 |
| 23 | 1 |
| 24 | 1 |
| 25 | 4 |
| 26 | 3 |
| 27 | 3 |
| 28 | 2 |
| 29 | 0 |
| 30 | 0 |
| 31 | 0 |
| 32 | 1 |
| 33 | 0 |
| 34 | 1 |
| 35 | 0 |
| 36 | 0 |
| 37 | 1 |
| 38 | 1 |
| 39 | 1 |
| 40 | 1 |
| 41 | 0 |
| 42 | 0 |
| 43 | 1 |

**Table S2 TANEC cases at different days of life**

| Days of life(day) | TANEC cases | Number of transfusion episodes |
| --- | --- | --- |
| ≤14 | 36 | 11967 |
| 15-28 | 53 | 10685 |
| 29-42 | 37 | 8853 |
| 43-56 | 18 | 5321 |
| >56 | 5 | 4811 |

**Table S3 TANEC cases at different PMA**

| PMA | TANEC cases | Number of transfusions |
| --- | --- | --- |
| ≤26 | 2 | 663 |
| 27-28 | 7 | 2249 |
| 29-30 | 13 | 5406 |
| 31-32 | 41 | 8439 |
| 33-34 | 47 | 8959 |
| 35-36 | 24 | 7855 |
| 37-38 | 10 | 4677 |
| >38 | 5 | 3389 |

**Table S4 Prognosis of TANEC infants.**

|  |  |  |  | **TANEC vs No-NEC** | | | | **TANEC vs UNTA-NEC** | | | |
| --- | --- | --- | --- | --- | --- | --- | --- | --- | --- | --- | --- |
| **Outcomes** | **TANEC**  **(N=149）** | **No-NEC**  **(N=15213)** | **UNTA-NEC (N=795)** | **Crude OR** | ***P* value** | **Adjust OR *** | ***P* value** | **Crude OR** | ***P* value** | **Adjust OR **** | ***P* value** |
| PVL (n, %) | 9(6.1) | 939 (6.2) | 47 (5.9) | 0.98(0.50-1.92) | 0.947 | 0.97(0.49,1.92) | 0.928 | 1.02(0.49-2.14) | 0.951 | 0.78(0.36,1.68) | 0.529 |
| Severe ROP (n, %) | 11(7.4) | 706 (4.6) | 34 (4.3) | 1.64(0.88,3.04) | 0.118 | 1.47(0.78,2.78) | 0.239 | 1.78(0.88-3.61) | 0.106 | 1.26(0.59,2.67) | 0.553 |
| Severe BPD (n, %) | 98(66.2) | 7183 (47.2) | 380 (47.8) | 2.15(1.53-3.02) | <0.001 | 2.03(1.41,2.91) | <0.001 | 2.10(1.46-3.03) | <0.001 | 1.76(1.18,2.62) | 0.006 |
| LOS (n, %) | 30(20.3) | 1661 (11.0) | 150 (18.9) | 2.06(1.37-3.08) | <0.001 | 2.06(1.37,3.09) | <0.001 | 1.08(0.70-1.68) | 0.718 | 1.14(0.73,1.80) | 0.563 |
| Death (n, %) | 29(19.6) | 1606 (10.6) | 114 (14.3) | 2.05(1.36-3.08) | <0.001 | 1.69(1.08,2.64) | 0.022 | 1.44(0.92-2.27) | 0.111 | 1.11(0.69,1.79) | 0.674 |

BPD, bronchopulmonary dysplasia; LOS, late-onset sepsis; ROP, retinopathy of prematurity; PVL, periventricular leukomalacia. * Adjust for gestational age, Placental transfusion, Age at first feed in days≤3, PDA; ** Adjust for gestational age, Endotracheal incubation during resuscitation, Age at first feed in days≤3, inborn、low 5-minute Apgar score ≤7、duration of antibiotic therapy in 1st 7days of life in days >4.
